# Pertaining to Insurance

**Published:** 1876-09

**Authors:** 


We have been assured that there is no amalgamation of the Union Mutual Life Insurance Co. of Maine and the United States Life Insurance Co. of New York intended by the present management of those companies, but that the interests of every policy holder, in both companies, will be carefully and fully protected. The officers of these companies are gentlemen of large experience and undoubted integrity, hence we can see no reason why the insured should be dissatisfied.

---

We can at present writing give no positive information as to the exact financial condition of the French Insurance Corporation, "La Caissé Generale," which has a branch office in Philadelphia, but it is said that it is not in good standing at home ; and the fact that it has avoided making the required deposit at Albany, and entered this State, as have other foreign companies, does not add much in its favor. We hope to be able to give more and fuller information relative to this company soon.

---

Our correspondent, T. H. Mc., has a policy in the North America Life Insurance Co. of N. Y., which he has been informed has suspended, and asks our advice in the matter. In reply we would say that the company has not suspended, but is reported, under sworn statement of its officers, as perfectly solvent, and our advice to our friend is to hold on to his policy. We have been informed that the officers, who are also interested in another company, are endeavoring to get the policy holders of the North America to change their policies for those of the other company. We would advise all, however, to refuse that offer, and hold on to their old policies. Any further information relative to this company can be had by addressing the Insurance Department at Albany, N. Y.

---

The Franklin Fire Insurance Co., of Philadelphia, is undoubtedly one of the best companies doing business in this country. Its losses are always honorably adjusted and promptly paid.

---

The stock of the following New York City Fire Insurance companies is selling below the par value, viz. : Arctic, Adriatic, Amity, Columbia, Commerce, Firemen's Fund, Gebhard, Guaranty, Hope, Lorillard, Metropolitan, N. Y. & Yonkers, Relief, Resolute, and Franklin.

---

The July dividends of Hartford Insurance Companies were as follows :

| | | |
|---|---|---|
| Ætna Fire Insurance Co. | 6 per cent. | $180,000 |
| Hartford Fire Insurance Co. | 10 per cent. | 100,000 |

| | | |
|---|---|---|
| Phœnix Fire Insurance Co. | 15 per cent | $90,000 |
| National Fire Insurance Co | 6 per cent | 30,000 |
| Oriental Fire Insurance Co | 3 per cent | 13,500 |
| Atlas Fire Insurance Co. (prob.) | 6 per cent | 12,000 |
| Connecticut Fire Insurance Co | 6 per cent | 30,000 |
| Traveler's Insurance Co. | 6 per cent | 36,000 |
| Railway Passengers' Assurance Co | 5 per cent | 15,000 |
| Other Life Insurance Co. | (estimated) | 115,000 |
| Steam Boiler Insurance Co | 5 per cent | 10,000 |

---

To R. S. H. we would say, the company to which you refer is unworthy of your confidence ; it is notoriously litiguous, and in the event of your death your heirs would doubtless have a lawsuit on hand if they attempted to collect the amount of your insurance. If you have only been insured in it one year, as you state, you had better let the policy lapse, and take out a new one in a company about which there is no doubt.

---

From returns made to the Commissioner of Insurance, at the close of the year 1875, it appears that the total assets of the fire companies of Hartford were at that time, in round numbers, $15,000,000, invested, the amounts being stated in even hundreds of thousands, as follows :

| | |
|---|---|
| Bank stocks | $3,500,000 |
| County, town, city, and district bonds | 2,000,000 |
| Railroad bonds | 2,000,000 |
| Real estate mortgages | 1,600,000 |
| Railroad stocks | 1,100,000 |
| United States bonds | 700,000 |
| State stocks | 400,000 |
| Trust companies | 200,000 |

"The remainder was in real estate, loans on call, cash, premiums, etc. The relative amounts of the different classes of securities held indicate the opinions entertained concerning them as investments ; income, security and convertability being considered. It should be borne in mind, however, that the insurance companies pay no taxes on bank stock, railroad bonds, and other securities taxable in the hands of individuals, and that the net income they derive from them is, therefore, about two per cent. greater than that which citizens of Hartford, holders of the same securities, would receive."

---

**Never patronize Cheap John** or co-operative **insurance companies** ; the **best article is the cheapest in the end.**

